# Bayesian Analysis of MicroScale Thermophoresis Data to Quantify Affinity of Protein:Protein Interactions with Human Survivin

**DOI:** 10.1038/s41598-017-17071-0

**Published:** 2017-12-01

**Authors:** Maria-Jose Garcia-Bonete, Maja Jensen, Christian V. Recktenwald, Sandra Rocha, Volker Stadler, Maria Bokarewa, Gergely Katona

**Affiliations:** 1 Department of Chemistry and Molecular Biology, University of Gothenburg, Gothenburg, Sweden; 2 Department of Medical Biochemistry, University of Gothenburg, Gothenburg, Sweden; 3 Department of Biology and Biological Engineering, Chemical Biology, Chalmers University of Technology, Gothenburg, Sweden; 4 PEPperPRINT GmbH, Rischerstrasse 12, 69123 Heidelberg, Germany; 5 Department of Rheumatology and Inflammation Research, The Sahlgrenska Academy at University of Gothenburg, Gothenburg, Sweden

## Abstract

A biomolecular ensemble exhibits different responses to a temperature gradient depending on its diffusion properties. MicroScale Thermophoresis technique exploits this effect and is becoming a popular technique for analyzing interactions of biomolecules in solution. When comparing affinities of related compounds, the reliability of the determined thermodynamic parameters often comes into question. The thermophoresis binding curves can be assessed by Bayesian inference, which provides a probability distribution for the dissociation constant of the interacting partners. By applying Bayesian machine learning principles, binding curves can be autonomously analyzed without manual intervention and without introducing subjective bias by outlier rejection. We demonstrate the Bayesian inference protocol on the known survivin:borealin interaction and on the putative protein-protein interactions between human survivin and two members of the human Shugoshin-like family (hSgol1 and hSgol2). These interactions were identified in a protein microarray binding assay against survivin and confirmed by MicroScale Thermophoresis.

## Introduction

Biomolecules interact and recognize one another transiently or more persistently following simple principles of chemical mass action. To understand the nature of interactions various experimental techniques have been developed to measure the dissociation constant (K_D_) between interacting biomolecules. Reliably detecting interactions between different biomolecules and estimating the binding affinities is essential prior to *in vivo* studies and also for drug development.

MicroScale Thermophoresis (MST) is an emerging technique to study molecular interactions and it has been growing in popularity over the last decade. It is based on the thermophoresis effect, which manifests itself as a directed movement of particles in a temperature gradient^1^. MST is a useful technique to analyze protein-protein, protein-DNA/RNA and protein-liposome interactions. It requires a small amount of material and permits almost unrestricted buffer composition^2–4^.

During a standard MST experiment the solutions of two molecules are mixed: one molecule has a fluorescence label while the other is unlabeled. After an appropriate incubation time the observed fluorophore tagged biomolecules distribute themselves into complexes and free components depending on their concentrations and the dissociation constant of the interaction. The interaction of a fluorescent biomolecule with other molecules may provoke changes in the molecular properties (size, hydration and charge) and affects its thermophoretic movement^1^. These changes are used to measure affinities and to calculate the dissociation constant, K_D_, down to the range of a few hundred picomolar (pM)^2^.

MST has the advantages of requiring only a small amount of sample and being relatively easy to automate the measurements compared to other techniques^4^. The steady state change in fluorescence level upon complete binding often only amount to a few percent of the total intensity. Even the slightest nuisance biophysical or biochemical process and sample handing error can result in an anomalous observations. The increased data acquisition rate also has to be complemented with robust, automated procedures to analyze the binding curves and to quantify the uncertainty of determined dissociation constants^5^. To fully realize the power of automation, the anomalous observations need to be treated without human influence. Standard non-linear least square (NLLSQ) regression methods require the identification and exclusion of these anomalous observations from the analysis. While experienced human observers often can identify and remove these observations correctly, this is a time consuming process and it is prone to human error. Here we employ machine learning techniques to automate the analysis and substitute subjective outlier rejection procedures. Our goal is to increase the objectivity of the analysis by taking into account as much of the available evidence as possible and to obtain useful information from an imperfect data.

Our biological test systems consist of survivin:borealin interaction which has already been well studied^6–8^ and putative interactions between survivin and Shugoshin (Sgo) proteins. Shugoshins regulate the function of the Chromosome Passenger Complex (CPC) and the progression of mitosis^9–11^.

Clinically, human survivin is a tumorigenesis marker and there is substantial evidence concerning its overexpression in different cancer types^12^. Recent reports shine new light on its function in autoimmune conditions such as rheumatoid arthritis^13,14^, psoriasis^15^ and multiple sclerosis^16^. Clinical relevance of Sgos is less explored and could be indirectly connected to the novel therapeutic interest in protein phosphatase 2 A (PP2A) agonists^17^.

Survivin is a small 16 kDa protein that belongs to the Inhibitor Apoptosis Protein (IAP) family. It consists of an N-terminal BIR (Baculovirus Inhibitor of apoptosis protein Repeat) domain and a long α-helical C-terminus^18^. In cell division, survivin is localized at the centrosomes and microtubules, controlling the assembly of CPC at the inner centromeres by binding to phosphorylated histone 3^11^. Survivin attaches to the other proteins of CPC by forming a ternary complex between survivin/borealin/INCENP^6,9^. Borealin plays an important role targeting other members of the CPC, correcting the kinetochore attachment errors and stabilizing the bipolar spindle in human cells^19^. X-ray crystallography studies have reported interaction between the N-terminal of borealin (1–142) and other CPC members, such as survivin^7^.

The assembly of CPC to the centromere activates Aurora B kinase that phosphorylates kinetochore substrates and coordinates the dis-attachment between kinetochores and microtubules, which pulls sister chromatids apart.

Sgos (Sgo1 and Sgo2) counteract these pulling forces. In human cells, Sgos protect the cohesin protein complex, which keeps sister chromatids together from cleavage; preventing premature loss of centromeric cohesion, missegregation and mitotic arrest. This occurs through its direct binding with the phosphatase PP2A^20^. Recently, functions of Sgos in spindle associated complex and kinetochore protection have been described. These functions are regulated by CPC and also are required for the centromeric localization of Aurora B. In fission yeast, Sgo2 promotes CPC recruitment to centromeres by Cdc2-dependent phosphorylation of the survivin subunit. Reciprocally, survivin has been shown essential for the enrichment of Sgo2 at centromeres. In humans, the N-terminus of hSgol1, but not hSgol2, mimics the phosphorylated N-terminus of histone 3, which is recognized by the BIR-domain of survivin. Thus, a competition between hSgol1 and phospho-H3 for survivin might regulate the recruitment of CPC to the centromere. The question whether or not there is a physical contact between Sgo1 and survivin is controversial. Direct contact between human survivin and hSgol1 was not detected by yeast two-hybrid system^21^. In contrast, survivin and Sgo1 appears to interact in fission yeast^22^ and supporting evidence is also available from biophysical and structural studies^11^. The nature of Sgo2 and survivin interaction is also unclear. Importantly, when Sgo2 is missing it has a stronger effect on survivin localization^10,23,24^.

In this work, we explore the effect of the experimental errors and the uncertainty of determined dissociation constant in MicroScale Thermophoresis experiments. In addition to simulations, we test the method on experimental data, which were obtained from the interaction between survivin and short linear motifs of borealin, hSgol1 and hSgol2. Finally, we point out similarities in the localization and sequence of the putative binding motif in hSgol1 and hSgol2, which suggest a common evolutionary origin.

## Results and Discussion

### Probabilistic inference from simulated data

When evaluating a new statistical method, it is better to use a different error model for generating simulated data than what is used for inference. That way one can test how well the methods perform when confronted with unexpected errors. Here, we introduce serial dilution concentration errors and add outliers even though the competing inference strategies (non-linear least-squares regression and probabilistic Bayesian inference) are not prepared for them.

To cover a large concentration range, serial dilution schemes are most commonly applied. The highest concentration of an interaction partner is stepwise diluted to the lowest concentration in the series. Each dilution step presumably depends on the ratio of two normally distributed random variables with non-zero mean. Therefore, the concentration errors are not expected to be normally distributed and the resulting concentration distribution is not even symmetric. Through the series of multiplication, the variance and the skewedness of the concentration distributions are gradually compounded (Equations 1 and 2).

Thus, the process generating the concentrations becomes a biased random walk. The dependence on the previous concentration means that the concentration series has a tendency to jump and it is expected to stabilize on consistently lower or higher concentrations than the targeted ones. In addition, the asymmetry of the distribution results in an increased fraction of generated ligand concentrations that are lower than the targeted ones, at the expense of higher-than-targeted concentrations leading to an overall bias. As long as the pipetting coefficient of variation (CoV) is less than 1% (i.e. the pipetting performance adheres to the ISO8655 standard), the deviation from the targeted concentration is negligible on the logarithmic scale. It is worth noting though that current MST instrumentation and practice allows up to 16 dilutions in a single measurement and that the bias starts to be noticeable if the CoV exceeds 5% even on the logarithmic scale.

**Figure 1:**
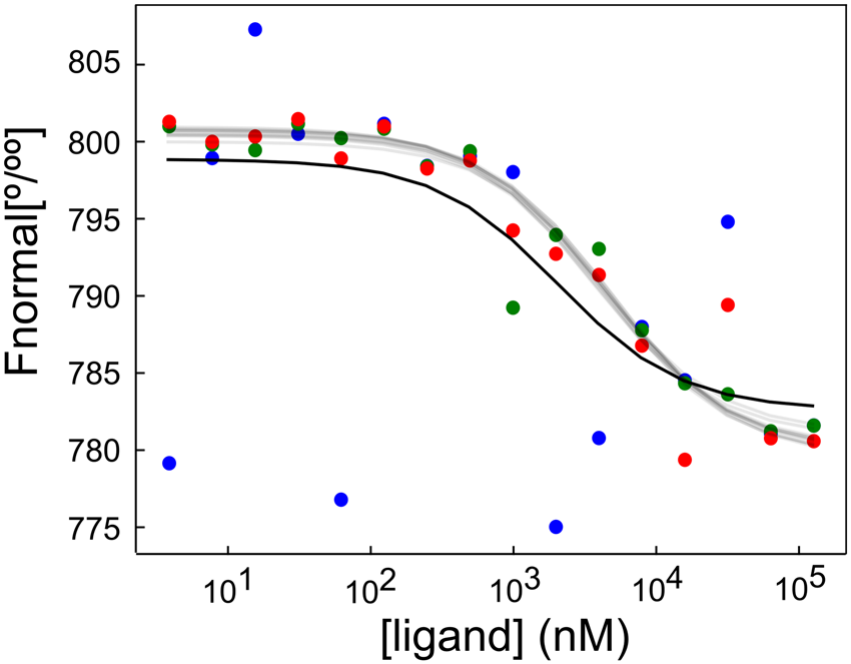
Bayesian analysis of simulated MST data. Simulated data set generated with the parameters of test case 1 (Table 1). Symbols with different colors belong to separate simulated dilution series. The thick line represents the NLLSQ fit to all data points whereas thin transparent lines show binding curves calculated using ten randomly selected posterior joint parameter sets.

Using the procedures described in the Material and Methods section we generated ten synthetic test cases with different known model parameters (Table 1; Fig. 1). The robust probabilistic model and a standard non-linear least squares regression (NLLSQ) method were tested against these cases. The results of the fits are summarized in Table 2. Figure 1 shows the simulated data of test case #1 and the inferred binding curves using the two methods. The probabilistic model is illustrated with a swarm of binding curves: each corresponding to a sample from the joint *a posteriori* probability distribution. Visually, this swarm captures the central tendency of the data better than the single point estimate binding curve determined by the NLLSQ method.

**Table 1:** Synthetic data generating parameters for the ten test cases.

#	K_D, true_ (μM)	B_true_	U_true_	Std. of ε	COV (μl)	Repeats
1	5.0	780	800	1	0.1	3
2	0.5	780	800	1	0.1	3
3	0.1	780	800	1	0.1	3
4	0.05	780	800	1	0.1	3
5	0.5	795	800	1	0.1	3
6	5.0	780	800	1	0.2	3
7	5.0	780	800	1	0.1	1
8	5.0	780	800	1	0.1	10
9	5.0	780	800	0.5	0.1	3
10	5.0	780	800	2	0.1	3

K_D,true_ is the known dissociation constant of the interaction, B_true_ and U_true_ represents the ideal fluorescent measurement of any type associated with the labelled protein with the ligand fully bound and ligand absent form, respectively. ε is the normally distributed random variable added to the ideal fluorescent measurement (mean parameter 0, standard deviation in the table). COV is the coefficient of variation of the ideal pipettes used for generating the ligand concentration series.

**Table 2:** Comparison of the Bayesian inference and NLLSQ fit.

#	Robust K_D_ (μM)*	NLLSQ K_D_ (μM)**	Robust B*	NLLSQ B**	Robust U*	NLLSQ U**
1	**4.4**	2.1	**780.1**	782.6	**800.5**	798.9
	(3.4–5.6)	(0.60–8.2)	(779.1–781.1)	(777.9–786.3)	(799.9–801.1)	(796.3–801.6)
2	**0.53**	0.54	**779.9**	781.8	**799.8**	796.4
	(0.39–0.69)	(0.14–2.0)	(779.0–780.9)	(778.9–784.6)	(798.9–800.6)	(793.6–799.6)
3	**0.15**	2.4	**779.6**	778.8	**798.7**	792.2
	(0.11–0.19)	(0.090–26.3)	(779.0–780.2)	(771.3–783.6)	(797.6–799.9)	(788.9–796.6)
4	**0.049**	0.053	**780.1**	785.4	**800.3**	794.9
	(0.031–0.065)	(0.000–1.5)	(779.7–780.6)	(782.3–788.3)	(798.9–801.7)	(789.3–802.6)
5	**0.57**	0.84	**795.2**	793.9	**799.5**	796.4
	(0.17–1.4)	(0.000–1000.0)	(794.7–795.7)	(0.0–1000.0)	(798.6–800.3)	(790.3–801.6)
6	**4.8**	3.5	**779.7**	778.8	**799.6**	797.5
	(3.2–6.4)	(0.57–21.7)	(778.1–781.5)	(770.3–783.9)	(799.1–800.2)	(794.3–800.9)
7	**5.1**	3.5	**780.3**	779.3	**799.7**	801.3
	(3.2–8.1)	(1.2–9.5)	(778.6–781.7)	(773.9–783.9)	(798.9–800.6)	(798.6–804.3)
8	**4.9**	4.6	**780.6**	783.3	**799.8**	796.9
	(4.2–5.8)	(1.9–11.3)	(779.7–781.4)	(780.3–785.9)	(799.5–800.1)	(783.3–798.3)
9	**4.8**	76.3	**780.5**	764.4	**800.2**	796.6
	(4.0–5.6)	(7.1–1000.0)	(779.6–781.2)	(0.0–782.6)	(799.8–800.5)	(794.3–798.9)
10	**7.7**	2.2	**779.3**	782.0	**798.8**	796.7
	(4.2–11.7)	(0.27–18.4)	(777.3–781.5)	(775.3–786.3)	(797.7–799.9)	(793.6–800.3)

Variable B and U represents the fluorescent measurement of any type associated with the labelled protein with the ligand fully bound and ligand absent form, respectively. K_D_ is a dissociation constant of the ligand and labelled protein. Bold results indicate estimates closer to the true value. *Median and HDI 95% interval of the sampled posterior probability distribution. **Maximum likelihood estimate and confidence interval (95%).

In all tested cases, the NLLSQ regression was less accurate than the probabilistic robust model (Table 2). The credible intervals (95% probability) of the probabilistic robust models always include the true K_D_ value. The same is true for the confidence interval (at 0.95 confidence level), but the confidence interval is always broader than the credible interval. In the test cases #2 and #5 the NLLSQ point estimate of K_D_ is very inaccurate and it misses the target by one order of magnitude. By comparing test case #1 and #6 one can conclude that concentration errors at realistic levels do not substantially increase to the uncertainty of K_D_. Test cases #1, #7 and #8 only differ in the number of repeated simulated experiments. Judging from the credible and confidence intervals, both type of fits get more accurate as the repeats increased, but the robust Bayesian approach requires less repeats to achieve the same level of precision. The credible interval narrows dramatically going from 1 to 10 repeats and the typical 3 repeats provide a good compromise between experimental requirements and accuracy of results. Similarly, more precise measurements would not benefit the NLLSQ method as the comparison of test cases #1, #9 and #10 shows. Quite the contrary, the NLLSQ method seems to perform best in test case #10 where the measurement error is the highest. In this case the contrast between the outliers and random variation around the true values is less apparent leading to less bias by the anomalous observations.

### Identification of survivin binding peptides by printed microarrays

**Figure 2:**
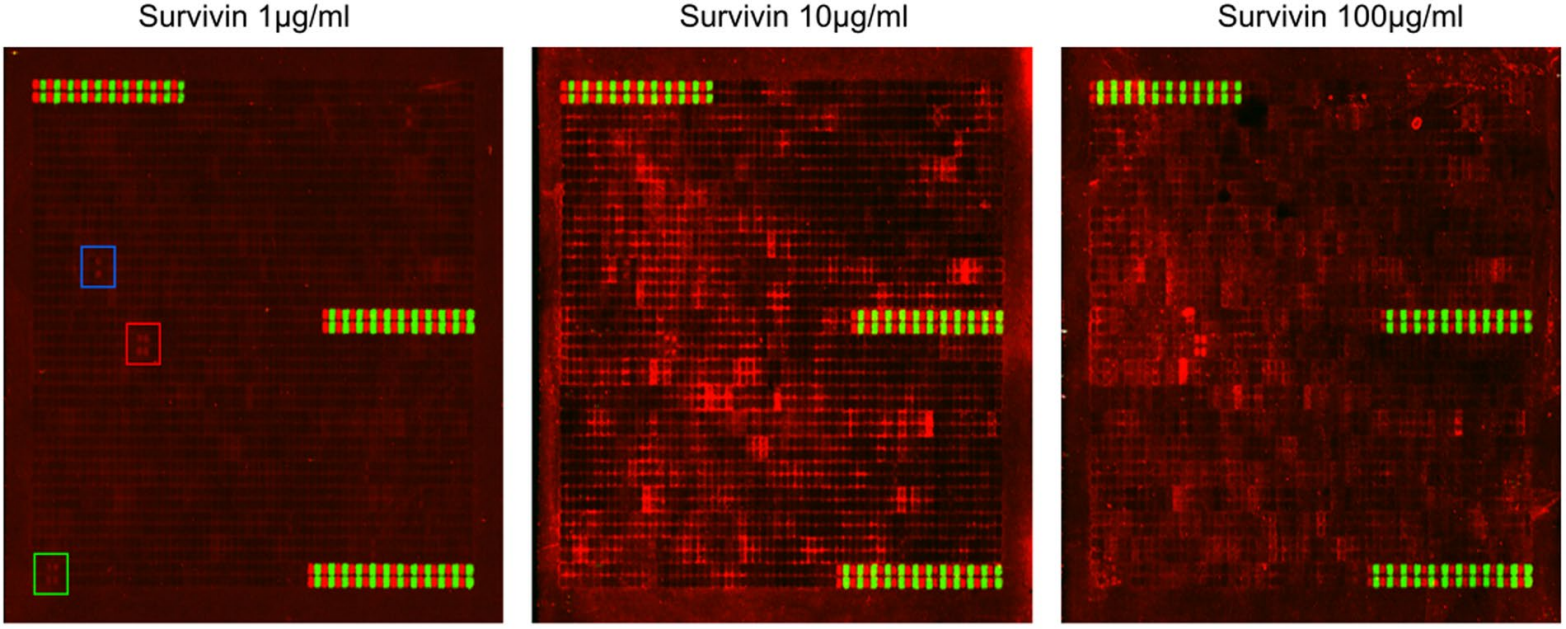
Binding pattern of human survivin to laser printed peptide microarrays at three survivin soaking concentration. The rectangles shows the duplicate overlapping peptides derived from borealin (blue), hSgol1 (red) and hSgol2 (green) sequence.

We tested the Bayesian inference method on potential binding interactions of survivin identified by PEPperCHIP Peptide Microarray (PEPperPRINT, GmbH, Heidelberg, Germany). These microarrays consisted of overlapping peptides originating from candidate proteins from the human proteome including hSgol1, hSgol2 and the N-terminal region of borealin (1–93). The peptides were generated by peptide laser printing^25^ (Fig. 2). Among the overlapping peptides, the main responses with the clearest spot morphologies were attributed to the basic hSgol1 peptides KREEKRKANRRKSKR and RKANRRKSKRMSKYK (and to some extent the adjacent DTQERKREEKRKANR peptide) and the hSgol2 peptides ECQVKKVNKMTSKSK and KVNKMTSKSKKRKTS. In addition, a peptide derived from borealin (GSSRVAKTNSLRRRK) also shows clear signal in the microarray at 1 μg/ml soaking concentration of survivin. The spots are consistently visible at 1, 10 and 100 μg/ml survivin concentrations in the assay buffer (Fig. 2). Quantitative analysis of the fluorescent intensities of the specific and control antibodies for borealin, hSgo1l and hSgol2 are shown in Supplementary Fig. S1, S2 and S3, respectively.

The consensus motif for hSgol1 and hSgol2 peptides is RKANRRKSKR and KVNKMTSKSK, respectively. Other basic peptides like SPTDPKACNWKKYKF, KTFRKVKDSSSEKKR, EVSKIVTVSTGIKKK or HQSSFLLSASKKKRI did not react with survivin in a similar manner. The identified part of hSgol1 (291–312) is overlapping with a KEN region^26^ which is targeted by the ubiquitin ligase activity of anaphase-promoting complex (APC) also known as cyclosome^27^. A similar pattern can be observed in the much larger hSgol2 isoform where the combined, identified region (1066–1085) is followed by two downstream KEN motifs (1115–1117 and 1173–1175).

We aimed to quantify the interaction using the MicroScale Thermophoresis technique. Synthetic peptides that combine the two identified peptides (of hSgol1 and hSgol2, respectively) to a single peptide were designed. In the case of hSgol1 the region was also extended to incorporate the entire KEN motif yielding the hSgol1 peptide sequence KREEKRKANRRKSKRMSKYKEN (291–312)^27^. For testing the hSgol2 interaction the hSgol2 ECQVKKVNKMTSKSKKRKTS sequence (1066–1085) was used. Longer N-terminal segments of borealin are not suitable as positive control in MST measurements, because they are not soluble in typical aqueous buffers. The choice of soluble borealin peptide, GSSRVAKTNSLRRRK (6–20), was motivated primarily by the microarray experiments and the prior knowledge about the structural details of borealin:survivin interaction^7^.

### MicroScale Thermophoresis experiments and robust inference of K_D_ values

**Figure 3:**
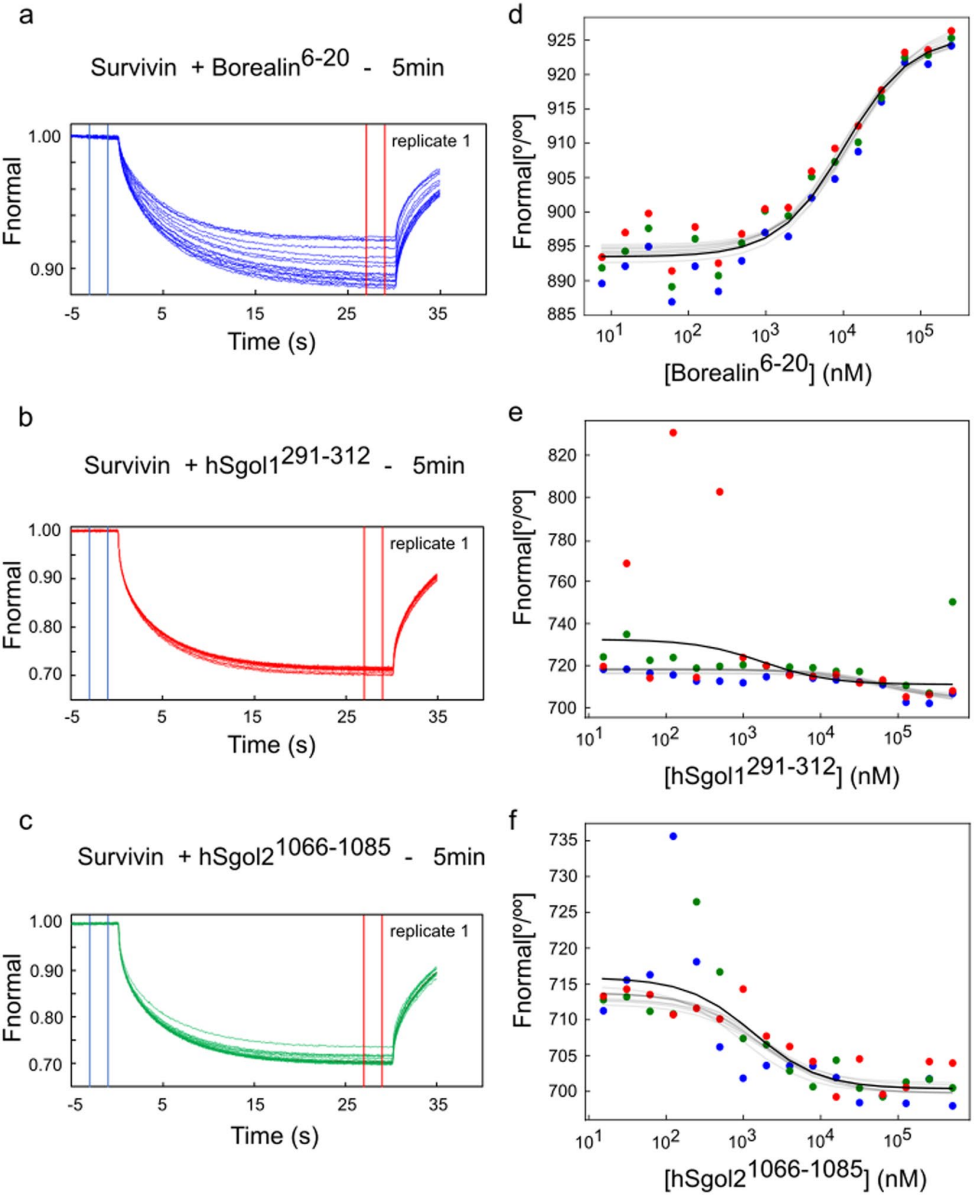
Bayesian analysis of experimental MST data. *Right:* Primary thermophoresis data (replicate 1) from serial dilutions of Borealin^6–20^ (**a**), hSgol1^291–312^ (**b**) and hSgol2^1066–1085^ (**c**) model peptide when incubated 5 min with fluorescent chemical labelled survivin, respectively. The cold (−3–−1s) and hot (27–29 s) regions (**a**–**c**) used to analyze the thermophoresis binding curves are represented by blue and red vertical lines, respectively (for replicates 2 and 3, respectively; see Supplementary Fig. S4). *Left:* Thermophoresis binding curves of Borealin^6–20^ (**d**) hSgol1^291–312^ (**e**) and hSgol2^1066–1085^ (**f**) interaction. The blue, green and red symbols represents measurements performed on three independent dilution series, respectively. The thick line represents the NLLSQ minimized binding curves, thin lines are ten random samples from the posterior distribution determined by the robust Bayesian procedure.

Primary MicroScale Thermophoresis data of borealin^6–20^, hSgol1^291–312^ and hSgol2^1066–1085^ is shown in Fig. 3. The replicates of the thermophoretic progress curves are displayed in the Supplementary material, Supplementary Fig. S4. The median of K_D_ posterior distribution for borealin^6–20^, hSgol1^291–312^ and hSgol2^1066–1085^ are 10.8 μM, 80.9 μM and 1.6 μM, respectively (Tables 3 and 4). The two NLLSQ fitting approach was performed with identical initial parameters (the initial parameters are listed displayed in the Supplementary material, Supplementary Table S1). For comparison the reported K_D_ of the N-terminal hSgol1 tetrapeptide is 6.2μM^11^. The MST binding curves between borealin^6–20^ and survivin in Buffer A have high amplitude and there are no outliers present. The NLLSQ and robust Bayesian fitting procedure infers comparable K_D_ values and the intervals of uncertainty are similar. On the other hand binding data of hSgol1^291–312^ in Buffer B is relatively poorer quality with several outliers present and we can expect a different fitting result. For human observers it is immediately obvious that certain observations such as 122 nM, 488 nM and 30 nM concentrations for hSgol1^291–312^ in replicate 3 (see Supplementary material, Supplementary Fig.S4) and 122 nM for hSgol2^291–312^ in replicate 1 (Fig. 3), do not fit into the pattern of a sigmoid binding curve. Longer incubation times increase the number of outliers even further (see Supplementary material, Supplementary Fig. S5 and S6 and Table S2). Commonly used NLLSQ method is particularly sensitive to outlier observations^28,29^. Using the robust Bayesian approach (Fig. 3) the extreme observations are a natural part of the distribution tails and have only a limited effect on the central tendency of the fit. When comparing the NLLSQ fits as implemented in the software PALMIST^30^ we see that the NLLSQ point estimates sometimes are not part of the Bayesian highest density intervals and the confidence intervals are broader than the Bayesian credible intervals (even if they answer very different questions) (Tables 3 and 4). Thus, we can establish that experimental data frequently include data points which, if not rejected, can cause substantial inaccuracy in K_D_ estimates when using NLLSQ minimization. To analyze which peptide (hSgol1^291–312^ or hSgol2^1066–1085^) has higher affinity to survivin, a binding affinity comparison is included in Supplementary material (Supplementary Fig. S7).

**Table 3:** Model parameters inferred from experimental data using the interactions of chemical labelled survivin with Borealin^6–20^ in Buffer A after 5 min incubation.

Borealin^6–20^			
	Robust*	NLSSQ**	Palmist***
K_D_ (μM)	10.8 (7.1–14.9)	10.2 (7.1–14.7)	10.0 (6.0–17.0)
∆G (kJ/mol)	−28.3 (−29.3–−27.5)	−28.4 (−29.3–−27.5)	−28.4 (−29.7–−27.1)
B	926.0 (923.7–928.4)	925.7 (923.3–928.3)	926.0 (922–930)
U	893.7 (892.3–895.0)	893.5 (892.3–894.6)	893.4 (891.6–895.2)

Variable B and U represents the T-jump + thermophoresis difference in F_normal_ for the fully bound ligand and unbound labelled protein, respectively. K_D_ is a dissociation constant of the ligand and labelled protein. *Median and HDI 95% interval of the sampled posterior probability distribution. **Maximum likelihood estimate and confidence interval (95%). ***Maximum likelihood estimate and confidence interval using ESP settings (95%) as determined by the software PALMIST^30^.

**Table 4:** Model parameters inferred from experimental data using the interactions of chemical labelled survivin hSgol1^291–312^ and hSgol2^1066–1095^ in Buffer B after 5 min incubation.

hSgol1^291–312^			
	Robust*	NLSSQ **	Palmist***
K_D_ (μM)	80.7 (12.2–398.2)	1.8 (0.058–131.0)	1.8 (n/a- n/a)
∆G (kJ/mol)	−23.3 (−28.0–−19.3)	−32.7 (−41.2–−22.1)	−32.7 (n/a-n/a)
B	703.0 (689.5–708.4)	711.1 (696.2–718.9)	711.0 (n/a- n/a)
U	717.8 (715.6–719.5)	732.5 (721.2–747.2)	730.0 (660.0–780.0)
**hSgol2** ^**1066–1085**^			
	Robust*	NLSSQ **	Palmist***
K_D_ (μM)	1.6 (0.72–3.0)	1.4 (0.55–3.5)	1.4 (0.40–5.8)
∆G (kJ/mol)	−33.0 (−34.9–−31.4)	−33.3 (−35.6–−31.0)	−33.3 (−36.4–−29.8)
B	700.2 (698.9–701.5)	700.4 (698.2–702.6)	700.0 (697.0–704.0)
U	713.3 (711.8–714.8)	715.9 (713.2–718.6)	716 (712.0–720.0)

Variable B and U represents the T-jump + thermophoresis difference in F_normal_ for the fully bound ligand and unbound labelled protein, respectively. K_D_ is a dissociation constant of the ligand and labelled protein. *Median and HDI 95% interval of the sampled posterior probability distribution. **Maximum likelihood estimate and confidence interval (95%). ***Maximum likelihood estimate and confidence interval using ESP settings (95%) as determined by the software PALMIST^30^.

### Putative binding region in Shugoshins

**Figure 4:**
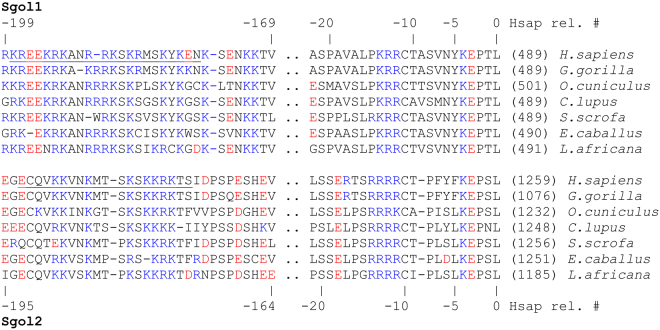
Multiple sequence alignment (MSA) of the Sgol C-terminal region. Positively and negatively charged residues are marked with blue and red colors, respectively. Numbers in parentheses refer to the residue number of the reference point in Sgol1 and Sgol2, respectively. This reference point is in a region that shows high sequence similarity and is most likely homologous even between Sgol1 and Sgol2. The reference point is the basis of relative numbering of hSgol1 and hSgol2 above and below the MSA. From this comparison it is apparent that the hSgol1^291–312^ and hSgol2^1066–1085^ peptide is about the same relative primary sequence distance from the C-terminal reference point. The hSgol1^291–312^ and hSgol2^1066–1085^ region is similar within Sgol1 and Sgol2 families, but between families the similarity is less obvious.

There is a subtle similarity between the two peptides (hSgol1^291–312^ and hSgol2^1066–1085^) in the consensus region (Fig. 4), which can be described by a hypothetic motif [EQ]-x-[KR]-K-[AV]-N-[KR]-x-x-S-K. This strict pattern can only be found in hSgol1 and hSgol2 in the human proteome (Uniprot). If we summarize the previously described binding N-terminal sequences^11^ (histone and hSgol1) with a strictly defined consensus motif (<x-A-[RK]-[TE]-[RK]) we still obtain 22 matches in the human proteome, but for example hSgol2 is absent due to an incompatible N-terminus with sequence MECPV. hSgol2 N-terminus is not detected even with a more permissive consensus motif (<x-[AV]-[RKH]-[TSE]-[RKE]) and the notion of specific N-terminal recognition is potentially problematic given the overwhelming evidence for physical survivin-hSgol2 interaction in fission yeast^10,24^.

The hSgol1^291–312^ and hSgol2^1066–1085^ peptides are both highly charged, but their structure does not appear to be identical with hSgol1^291–312^ showing signs of increased local order (see Supplementary Result and Discussion and Fig. S8). The sequence conservation in the Sgol1^291–312^ and Sgol2^1066–1085^ region is strong within mammalian Sgol1s and Sgol2s, respectively (Fig. 4). On the other hand, it is uncertain whether the subtle similarity between mammalian Sgol1^291–312^ and Sgol2^1066–1085^ regions results from true homology or merely converging parallel evolution. It is worth noting that there are approximately the same number of residues between the evolutionally conserved C-terminal domain and Sgol1^291–312^ and Sgol2^1066–1085^ regions, respectively (Fig. 4). This must be interpreted in the light that the length of Sgol2 in different species varies greatly, even when compared to our close mammalian relative, gorillas. Another way to judge evolutionary conservation is to compare with other recognizable features for example the KEN box^27^, which appears to be only present in humans (and only in hSgol1 not in hSgol2) in this (limited) comparison. Nevertheless, the binding locations relative to the C-terminal domain appears to be remarkably constant and may play a role in specific positioning of survivin. This suggests a functional link between survivin and the hSgol C-terminal domain.

Thus, the new motif in Shugoshin-like proteins appears to show signs of evolutionary conservation/convergence and may be recognized by survivin more specifically. Since this motif is not located at the N-terminus of Shugoshin-like proteins it does not immediately exclude the possibility of simultaneous binding to phosphorylated histones.

## Conclusion

We described a robust Bayesian inference protocol that minimizes bias and allows unsupervised high-throughput MST analysis. This method is sufficiently fast and highly tolerant to frequently observed outlier data points. The resulting *a posteriori* probability distributions immediately provide the uncertainty of the estimates, which can be directly used for Bayesian A/B testing.

We also reported a putative interaction footprint of survivin on hSgol1 and hSgol2. This evidence adds a piece to the intriguing puzzle about how the chromosome passenger complex is formed and acts in concert with the sister chromatid cohesion apparatus.

## Materials and Methods

### Protein production and purification

Survivin was expressed in *E.coli* BL21(DE3) star (Merck) cells using the pHIS8 expression vector used by Verdecia *et al*.^18^ The expression was induced at OD600 0.6–0.8 in Luria Bertani (LB) media by 0.5 mM IPTG and incubated during 3–4 h at 30 °C. The cells were harvested at 6000 g during 20 min and resuspended in lysis buffer containing 50 mM Tris pH 8, 500 mM NaCl, 10 mM imidazole, 0.25 mM Pefabloc®SC (Sigma), 20 mg/ml DNAse (Sigma) and 0.2 mg/ml Lysozyme (Sigma). The cells were lysed with a high pressure homogenizer, using 3 cycles of 15,000–20,000 bar. Survivin was purified by Ni^2+^ chelation chromatography using 5 ml HisTrap FF column (GE Healthcare). Overnight dialysis in 50 mM Tris pH8, 150 mM NaCl and 1 mM DTT buffer and a gel filtration purification using a Superdex 75 100/300GL column (GE Healthcare) were done before chemical labelling. The SDS PAGE of purified survivin before and after chemical labelling is displayed in Supplementary Fig. S9. The borealin^6–20^, hSgol1^291–312^ and hSgol2^1066–1085^ peptides were chemically synthetized (Genscript).

### Peptide microarrays

Pre-staining of one of the PEPperCHIP^®^ Peptide Microarrays was done with the secondary 6X His Tag Antibody DyLight680 antibody at a dilution of 1:1000 and with monoclonal anti-HA (12CA5)-DyLight800 control antibody at a dilution of 1:1000 to investigate background interactions with the protein-derived peptides that could interfere with the main assays. Subsequent incubation of other peptide microarray copies with survivin at concentrations of 1 µg/ml, 10 µg/ml and 100 µg/ml in incubation buffer was followed by staining with the secondary 6X His Tag Antibody DyLight680 (Rockland Immunochemicals) antibody and the monoclonal anti-HA (12CA5)-DyLight800 control antibody (Rockland Immunochemicals) as well as by read-out at scanning intensities of 7/7 (red/green). HA and His tag control peptides were simultaneously stained as internal quality control to confirm the assay quality and to facilitate grid alignment for data quantification. Read-out was performed with a LI-COR Odyssey Imaging System, while quantification of spot intensities and peptide annotation were done with PepSlide^®^ Analyzer. Quantification of spot intensities and peptide annotation were based on the 16-bit gray scale tiff files at scanning intensities of 7/7 that exhibit a higher dynamic range than the 24-bit colorized tiff files.

### MicroScale Thermophoresis

MicroScale Thermophoresis experiments were performed according to the NanoTemper technologies protocol in a Monolith NT.115 (green/blue) instrument (NanoTemper Technologies). Serial dilutions with borealin^6–20^ (GSSRVAKTNSLRRRK) were done using 50 mM Tris pH 8.0, 150 mM NaCl, 1 mM DTT buffer with 0.05% Tween (Buffer A).

Survivin was labelled with the MO-L005 Monolith™ Protein Labeling Kit GREEN-MALEIMIDE (Cysteine Reactive) from NanoTemper Technologies. Peptides derived from hSgol1^291–312^ (KREEKRKANRRKSKRMSKYKEN) and hSgol2^1066–1085^ (ECQVKKVNKMTSKSKKRKTS) were analyzed in Buffer B (50 mM Tris pH 7.4, 150 mM NaCl, 10 mM MgCl_2_, 0.05% Tween-20 and 1 mM DTT). All samples were measured after an incubation of 5 min (according to “User Starting Guide Monolith® NT.115”).

The experiments were performed using 20% and 40% MST power and between 20–80% LED power at 24 °C. The MST traces were recorded using the standard parameters: 5 s MST power off, 30 s MST power on and 5 s MST power off. The reported measurement values are the combination of two effects: the fast, local environment dependent responses of the fluorophore to the temperature jump and the slower diffusive thermophoresis fluorescence changes.

### Probabilistic modelling of binding curves and maximum likelihood estimates

The probabilistic models were implemented in python with the help of modules numpy, scipy and pymc3.

A probabilistic model was used to simulate the concentrations in a serial dilution experiment. Each step in the series involves two pipetting events: the previous solution of the series (or the stock) is pipetted and the fresh buffer without the solute which results in a concentration dilution. For microscale thermophoresis experiments (and generally serial dilution experiments) it is recommended to use equal pipetting volumes to minimize the pipette specific systematic differences at different targeted volumes. Thus a reasonable *a priori* belief is that the variation in the concentration at each dilution step can be described by two normally distributed pipetting events with an assumed mean of the targeted volume and equal variance. The latent concentrations in the dilution series can be thus described with the following equations:

1 $${c}_{starting}\cdot (\frac{{N}_{a,1}}{{N}_{a,1}+{N}_{b,1}})\cdot (\frac{{N}_{a,2}}{{N}_{a,2}+{N}_{b,2}})\cdot ...\cdot (\frac{{N}_{a,n}}{{N}_{a,n}+{N}_{b,n}})$$

or

2 $${c}_{starting}\cdot \,\frac{1}{(1+\frac{{N}_{b,1}}{{N}_{a,1}})\cdot (1+\frac{{N}_{b,2}}{{N}_{a,2}})\cdot ...\cdot (1+\frac{{N}_{b,n}}{{N}_{a,n}})},$$

where *n* is the number of dilution steps *N*_*a*_ and *N*_*b*_ are random normal variables with the mean of the targeted pipetted volume of the ligand solution and the buffer solution respectively. The mean and standard deviation of the random variables are linked through the coefficient of variation of the pipette.

The targeted concentrations are part of geometric series:

3 $${c}_{starting}\cdot {(\frac{1}{2})}^{n}$$

The concentration dependence of the binding is described by the law of mass action:

4 $$U+(B-U)\cdot \frac{{c}_{fl}+c+{K}_{D}-\sqrt{{({c}_{fl}+c+{K}_{D})}^{2}-4{c}_{fl}c}}{2{c}_{fl}}+\varepsilon $$

where U and B are the measured values (T-jump, thermophoresis fluorescence difference or fluorescence yield directly whichever carries binding information) corresponding to the 100% unbound and bound ligand species, *c*_*fl*_ is the concentration of the fluorescent partner molecule and *K*_*D*_ is the dissociation constant. ε is the error of the measurement, which is free of dilution error i.e. equivalent to the error at the stock ligand concentration.

ε is assumed to be normally distributed for generating synthetic data and to account for the frequently observed “outliers” 20% of the synthetic data are replaced by samples from a uniform distribution that includes the U-B range.

In order to infer the *K*_*D*_ of the interaction we constructed an *a priori* probabilistic model using the Python library *pymc3* ^31^:

5 $$p({K}_{D}|lower=1,upper={10}^{6})=\frac{1}{upper-lower}$$

6 $$p(U|lower=0,upper=1000)=\frac{1}{upper-lower}$$

7 $$p(B|lower=0,upper=1000)=\frac{1}{upper-lower}$$

8 $$p({c}_{fl}|\mu ={c}_{fl,true},\tau =10{c}_{fl,true},lower=0,upper={10}^{5})=\sqrt{\frac{\tau }{2\pi }}{e}^{\frac{-\tau }{2}{({c}_{fl}-\mu )}^{2}};{c}_{fl}\in [lower,upper]$$

9 $$p(\nu |lower=1,upper=30)=\frac{1}{upper-lower};\nu \in [lower,lower+1,\mathrm{..}.,upper]$$

10 $$p(\lambda |lower=0,upper=100)=\frac{1}{upper-lower}$$

where *τ* is the precision parameter of the normal prior distribution of the fluorescent labeled protein concentration (reciprocal of the variance). The other parameters believed to be uniform and when the random variables of *K*_*D*_ *, U, B, c*_*fl*_ *, ν, λ* are outside of their bounds, their *a priori* probability is zero.

The probability distribution of variable *D(c)* corresponds to the thermophoretic/fluorescence yield observations at concentration *c* and is the only fixed parameter in the model:

11 $$p(D(c)|\mu =U+(B-U)\cdot \frac{{c}_{fl}+c+{K}_{D}-\sqrt{{({c}_{fl}+c+{K}_{D})}^{2}-4c{c}_{fl}}}{2{c}_{fl}},{\lambda }{,}{\nu })=\frac{{{\rm{\Gamma }}}^{\frac{\nu +1}{2}}}{{{\rm{\Gamma }}}^{\frac{\nu }{2}}}\sqrt{(\frac{\lambda }{\pi \nu })}{[1+\frac{\lambda {(D(c)-\mu )}^{2}}{\nu }]}^{-\frac{\nu +1}{2}}$$

where, *μ*, *ν* and *λ* corresponds to the location, degrees of freedom and scale parameter of the Student-T distribution. The Student T-distribution is assumed here because it tends to be robust against outlier observations and expected to capture the central tendency of D more efficiently.

To sample 5000 MCMC (Metropolis^32^ and No-U-Turn sampling (NUTS)^33^ iterations in a single chain on a Linux workstation (i7-3970X CPU at 3.50 GHz clock frequency) takes only one minute at most, which is at least one order of magnitude less time than performing a standard MST experiment.

The standard least squares regression was done by minimizing the squared residual between the observed data and prediction based on the law of mass action function ${(D(c)-f(c,U,B,{K}_{D}))}^{2}$ using the scipy.minimize.leastsq function call which uses the MINPACK library implementation of the Levenberg-Marquardt minimization algorithm^34,35^.

The confidence interval of the least squares estimates of K_D_, B and U parameters were determined from the minimum and maximum values of the fixed parameter p for which

12 $$RSS(p) < RSS(1+\frac{F}{N-P})$$

holds. RSS stands for the residual sum of squares differences between the data and the model in Eq. 4. RSS and RSS(p) are minimized for all parameters and for all parameters except p, respectively. N denotes the number of data points, and P is the number of parameters. F is the F-distribution value, calculated for α level of 0.05 and the degrees of freedom 1 and N − P.

For the NLLSQ evaluation of the experimental thermophoresis progress curves we also used the software PALMIST^30^ with the following options: Thermophoresis + T-jump, normalized fluorescence and default starting parameters. We used the same starting parameters in our NLLSQ calculation (see Supplementary Table S1). For estimating the confidence intervals in PALMIST we used the ESP method (0.95 confidence level). The binding free energy are calculated using the van’t Hoff formula: ΔG = RT ln K_D_ where T is 297.15 K and R is the universal gas constant.

## Electronic supplementary material

Supporting online text
