# Supplementary material for: Bayesian Analysis of MicroScale Thermophoresis Data to Quantify Affinity of Protein:Protein Interactions with Human Survivin
Source: Sci Rep. 2017 Dec 1;7:16816. doi: 10.1038/s41598-017-17071-0 (PMC5711809; doi:10.1038/s41598-017-17071-0)
Supplement: Supplementary file 1 — Supporting online text [file 41598_2017_17071_MOESM1_ESM.pdf]

Supporting online text

Bayesian Analysis of MicroScale Thermophoresis Data to Quantify Affinity of Protein:Protein Interactions with Human Survivin.

Maria-Jose Garcia-Bonete[1], Maja Jensen[1], Christian V. Recktenwald[2], Sandra Rocha[3], Volker Stadler[4], Maria Bokarewa[5] and Gergely Katona[1,*]

[1] Department of Chemistry and Molecular Biology, University of Gothenburg, Gothenburg, Sweden.
[2] Department of Medical Biochemistry, University of Gothenburg, Gothenburg, Sweden.
[3] Department of Biology and Biological Engineering, Chemical Biology, Chalmers University of Technology, Gothenburg, Sweden
[4] PEPperPRINT GmbH, Rischerstrasse 12, 69123 Heidelberg, Germany.
[5] Department of Rheumatology and Inflammation Research, The Sahlgrenska Academy at University of Gothenburg, Gothenburg, Sweden.

***Corresponding author:** Gergely Katona, Department of Chemistry and Molecular Biology, University of Gothenburg, Box 462, 40530 Gothenburg, Sweden; e-mail: gergely.katona@cmb.gu.se; phone: +46 31 7863959; fax: +46 31 7863910.

Supplementary Result and Discussion

MST experiments performed after two hours incubation

Different incubation times, buffer conditions and labelling approach were tested to evaluate the robustness of binding interactions between survivin and the peptides derived from hSgol isoforms. Two hours incubation time affected adversely the sample quality; which could be explained by the lack of long term thermal stability at room temperature or to sensitivity to oxidation as level of DTT in the buffers decreases over time (see Supplementary Fig.S5 online).

Comparison of binding affinities

Binding affinities need to be frequently compared to one another, for example we may ask the question if hSgol1$^{291\text{-}312}$ or hSgol2$^{1066\text{-}1085}$ peptide has higher affinity to survivin or they have practically the same affinity or we just do not have enough evidence to decide.

Firstly, one has to decide what we can call practical equivalence, or more specifically define a region of practical equivalence (ROPE).[1] A suitable lead may be to compare the binding free energy to the energy fluctuations at the experimental temperature (24ºC). The kT at 24ºC is 2.7kJ/mol and if the binding free energy difference is less than that the changes in the bound and unbound species are more dominated by random thermal energy fluctuations than any possible specificity difference between hSgol1$^{291\text{-}312}$ and hSgol2$^{1066\text{-}1085}$ to survivin.

In Supplementary Fig. S7a, we compare the distribution of ΔΔG to the ROPE (+/- 2.7kJ/mol) when the conditions are identical (after 5min incubation). We see that the highest density interval (95% probability) of ΔΔG does not overlap with the ROPE thus the available evidence is enough to support a potential claim that hSgol1$^{291\text{-}312}$ peptide has lower affinity to survivin than hSgol2$^{1066\text{-}1085}$.

Supplementary Fig. S7b shows the *a posteriori* probability distribution of the independent binding free energies for the hSgol1$^{291\text{-}312}$:survivin and hSgol2$^{1066\text{-}1085}$:survivin interactions when measured under identical labelling and buffer conditions (Buffer B) after 5min incubation.

Circular dichroism comparison of hSgol1$^{291\text{-}312}$ and hSgol2$^{1066\text{-}1085}$

The far-UV CD spectrum of hSgol1$^{291\text{-}312}$ in water and 0.05% Tween-20 shows a small positive shoulder at 220nm and a negative band at 197nm, which are characteristics of unordered structures (see Supplementary Fig. S8 online). The spectrum could also indicate locally ordered extended helical structures (known as polyproline (pPII) conformation) as characteristic pPII

CD spectra of peptides with low proline content have a distinct asymmetric couplet with a large negative band at about 195nm and small positive maximum at 218nm.[2,3] In phosphate buffer, at pH8, the peptide shows a similar spectrum, but the band at 197nm is slightly shifted to higher wavelengths and smaller in magnitude and the shoulder becomes negative, indicating a small conformational change. The spectra of hSgol2$^{291\text{-}312}$ peptide are characteristic of unordered structure (see Supplementary Fig. S8 online) and do not show the typical positive CD signal for pPII at 218nm.

Supplementary Materials and Methods

Circular dichroism spectroscopy

Far-UV circular dichroism (CD) spectra of hSgols peptides (50μM) in 20mM phosphate buffer pH8 or water with 0.05% Tween-20 were recorded using a Chirascan CD spectrometer (Applied Photophysics) in 1mm quartz cuvette at 10°C, with 1nm step size, a bandwidth of 1nm and a time-per-point of 0.8s. The spectra were averaged 5 times and baseline subtracted. The CD data are reported as mean residue ellipticity (degrees M^{-1} m^{-1}).

Table S1 Initial parameters for binding curve fitting according to the software PALMIST[3].

	**Unbound (Fnormal[°/°°])**	**Bound (Fnormal [°/°°])**	**K_D (nM)**	**[Fluorophore] (nM)**	**Cold area (s)**	**Hot area (s)**
Borealin$^{6-20}$*	889	924	500	500	-3 – -1	27-29
hSgol1$^{291-312}$_5min**	718	706	500	20	-3 – -1	27-29
hSgol2$^{1066-1085}$_5min**	711	698	500	20	-3 – -1	27-29
hSgol1$^{291-312}$_120min**	719	718	500	20	-3 – -1	27-29
hSgol2$^{1066-1085}$_120min**	774	708	500	20	-3 – -1	27-29

* Experimental data obtained from the interaction of chemical labelled survivin with Borealin$^{6-20}$ in buffer A after 5min incubation at 24ºC.

** Experimental data obtained from the interaction of chemical label survivin with hSgol1$^{291-312}$ and hSgol2$^{1066-1085}$ in buffer B after 5 and 120min incubation at 24ºC.

Table S2 Model parameters inferred from experimental data using chemical labelled survivin with hSgol1$^{291\text{-}312}$ and hSgol2$^{1066\text{-}1085}$ interaction in buffer B and incubation of 120min. Variable B and U represents the fluorescent readout of any type associated with the fully bound ligand and unbound labelled protein, respectively. K_D is a dissociation constant of the ligand and labelled protein.

Survivin + hSgol1$^{291\text{-}312}$ 120min

	Robust *	NLSSQ **	Palmist ***
K_D (μM)	53.6 (0.027-699.4)	7.8 (0.24-112.5)	8.0 (0- n/a)
ΔG (kJ/mol)	-24.3 (-43.1 – -18.0)	-29.1 (-37.7 – -22.5)	-29.0 (n/a-n/a)
B	702.8 (680.1-713.3)	707.7 (698.2-714.2)	708.0 (669.0-717.0)
U	715.9 (713.0-718.7)	722.8 (718.2-728.9)	723.0 (715.0-739.0)

Survivin + hSgol2$^{1066\text{-}1085}$ 120min

	Robust *	NLSSQ **	Palmist ***
K_D (μM)	1.6 (0.045-599.8)	0.0 (0.0-0.013)	0.0 (n/a- n/a)
ΔG (kJ/mol)	-33.0 (-41.8 – -18.3)	n/a (n/a – -44.9)	n/a (n/a-n/a)
B	703.2 (697.9-706.8)	710.5 (701.6-719.2)	711.0 (697.0-723.0)
U	710.6 (706.2-713.8)	950.6 (769.6-1000.0)	1000.0 (700.0-1200.0)

* Median and HDI 95% interval of the sampled posterior probability distribution.
** Maximum likelihood estimate and confidence interval (95%).
*** Maximum likelihood estimate and confidence interval using ESP settings (95%) as determined by the software PALMIST[4].

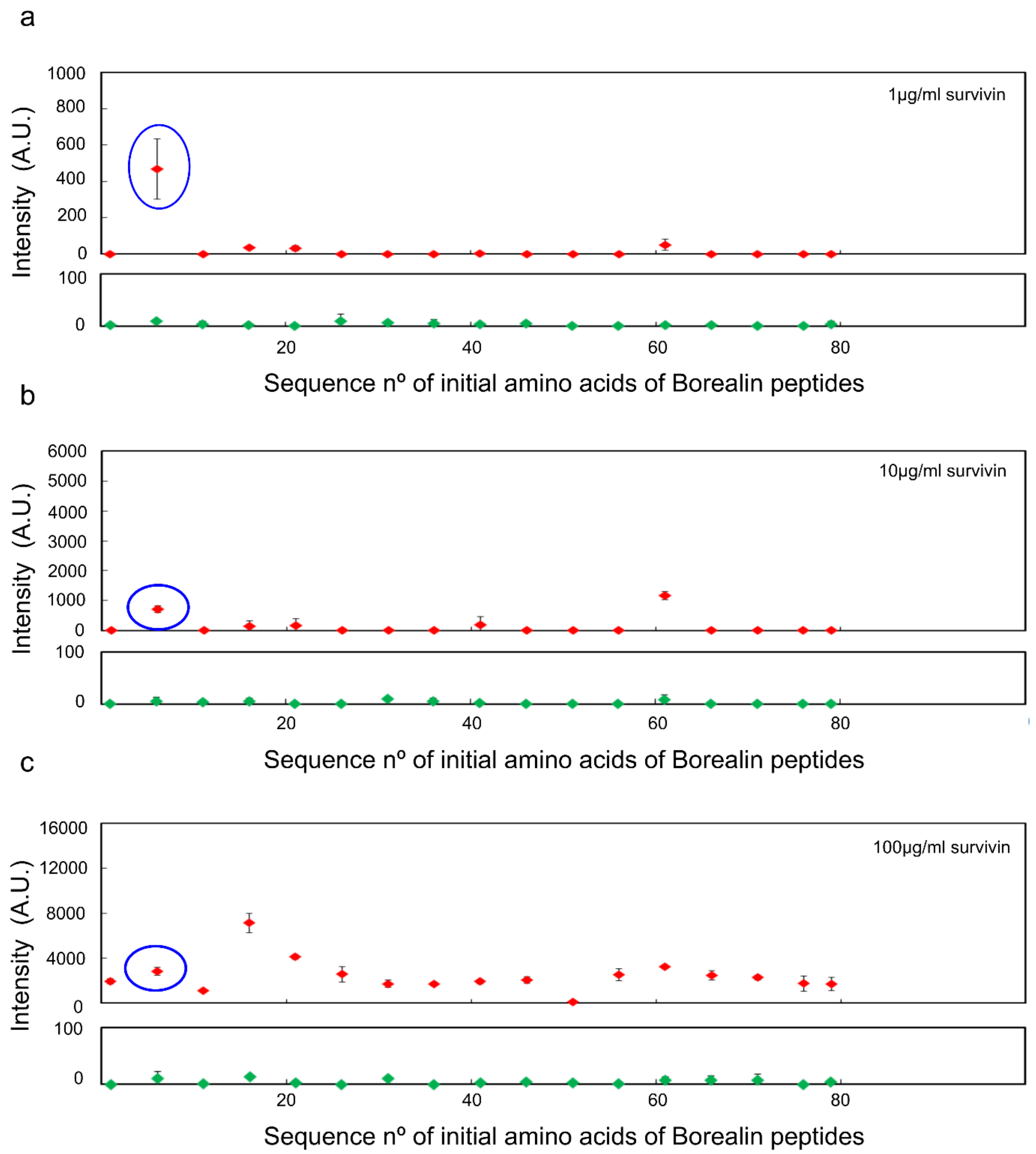

Figure S1 Microarray intensity plot from N-terminal Borealin peptides. Three different concentration of survivin are tested: 1µg/ml (a), 10µg/ml (b) and 100µg/ml (c). The intensity is represented as the mean of two replicates and the error bars represent the standard deviation around the mean. Red intensity plot represents the secondary 6X His-tag Antibody DyLight680 used for survivin detection. Green intensity plot represents the monoclonal anti-HA (12CA5)-DyLight800 used as a microarray control. In panels b and c the noise level is higher than in panel a.

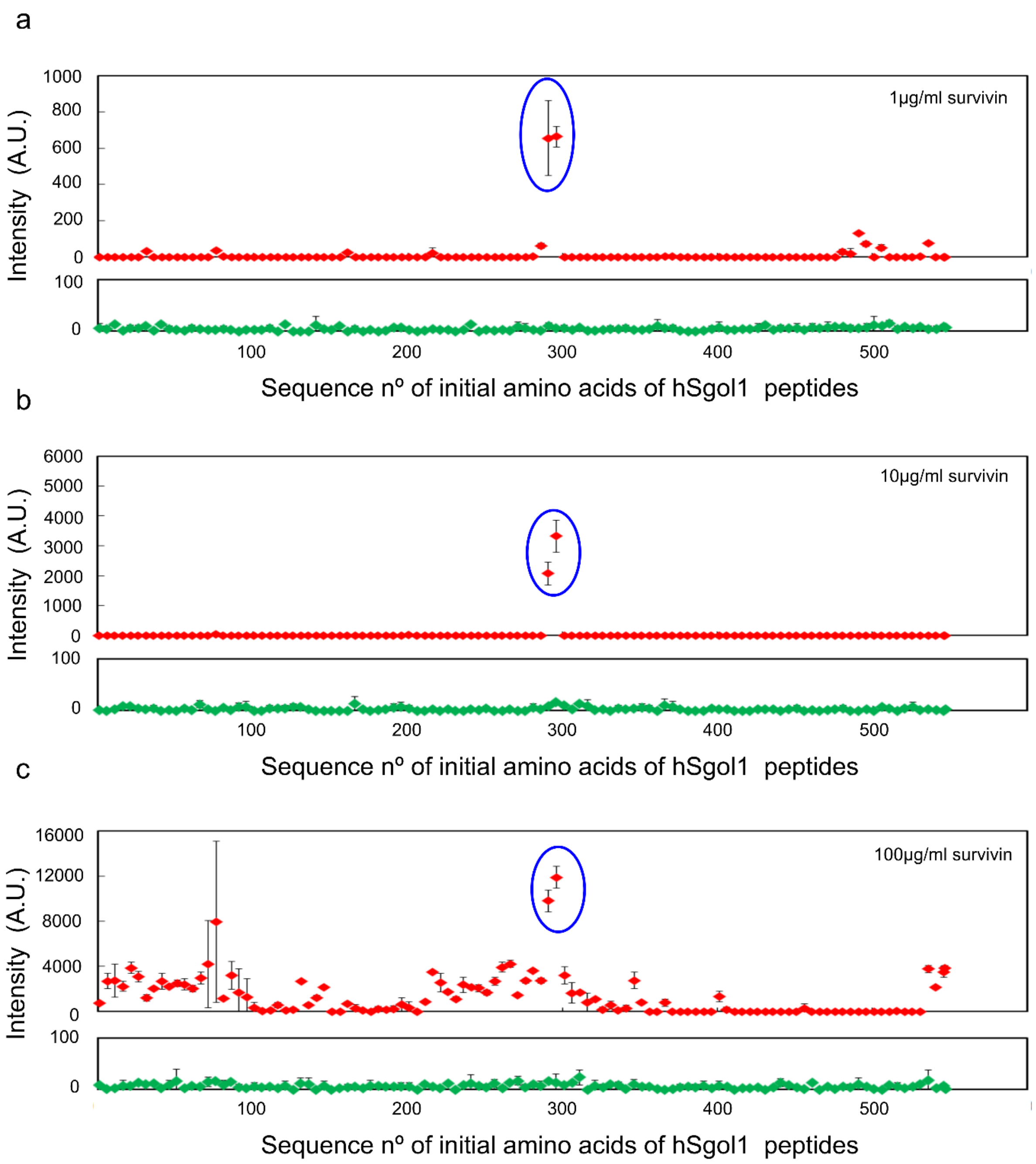

Figure S2. Microarray intensity plot from hSgol1 peptides. Three different concentrations of survivin are tested; 1µg/ml (a), 10µg/ml (b) and 100µg/ml (c). The intensity is represented as the mean of two replicates and the error bars represent the standard deviation around the mean. Red intensity plot shows the secondary 6X His-tag Antibody DyLight680 used for survivin detection. Green intensity plot indicates the monoclonal anti-HA (12CA5)-DyLight800 used as a microarray control. In panels a and b the noise level is lower than in panel c.

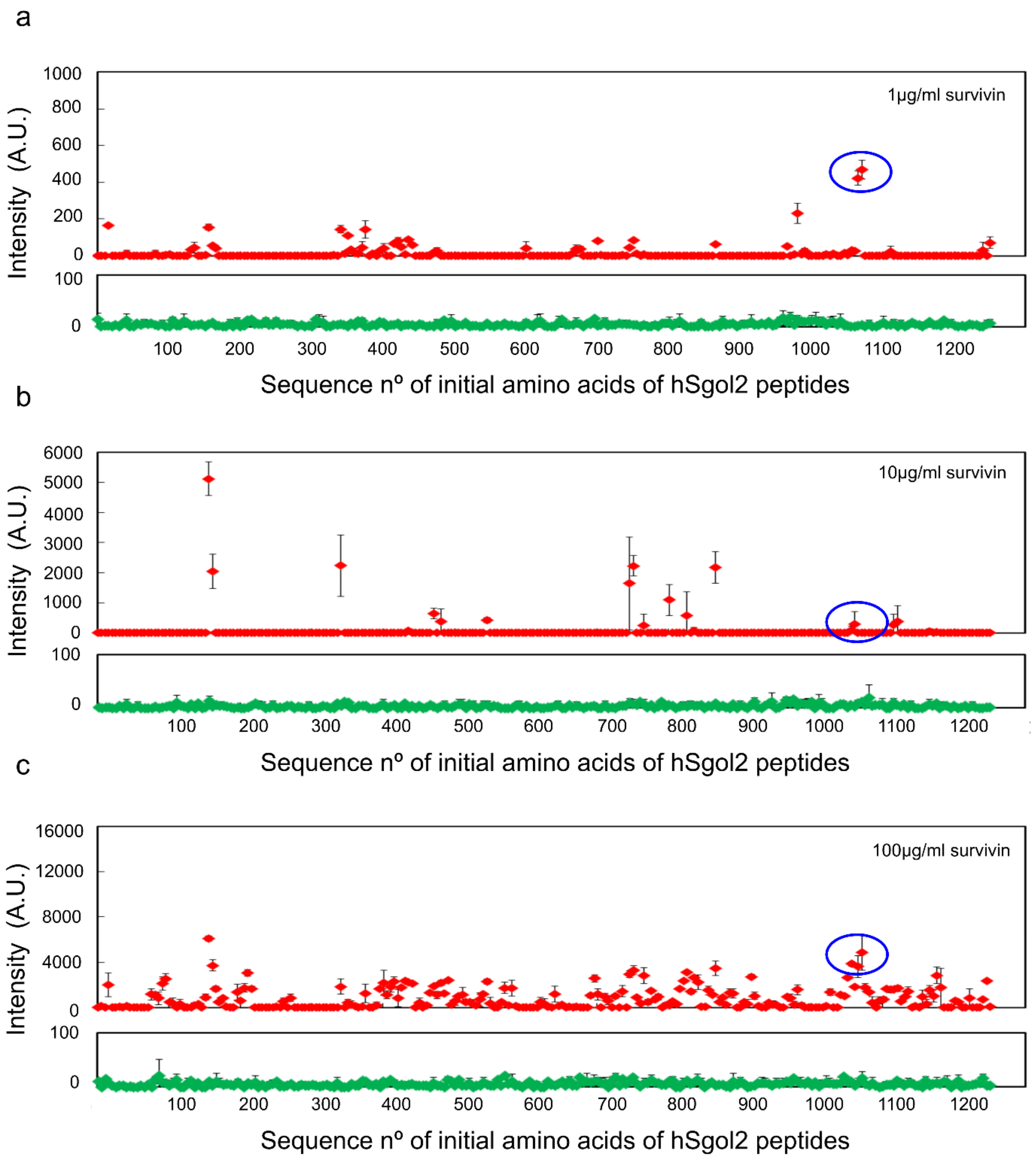

Figure S3. Microarray intensity plot from hSgol2 peptides. Three different concentration of survivin are tested: 1µg/ml (a), 10µg/ml (b) and 100µg/ml (c). The intensity is represented as the mean of two replicates and the error bars represent the standard deviation around the mean. Red intensity plot represents the secondary 6X His-tag Antibody DyLight680 used for survivin detection. Green intensity plot represents the monoclonal anti-HA (12CA5)-DyLight800 used as a microarray control. In panels b and c the noise level is higher than in panel a.

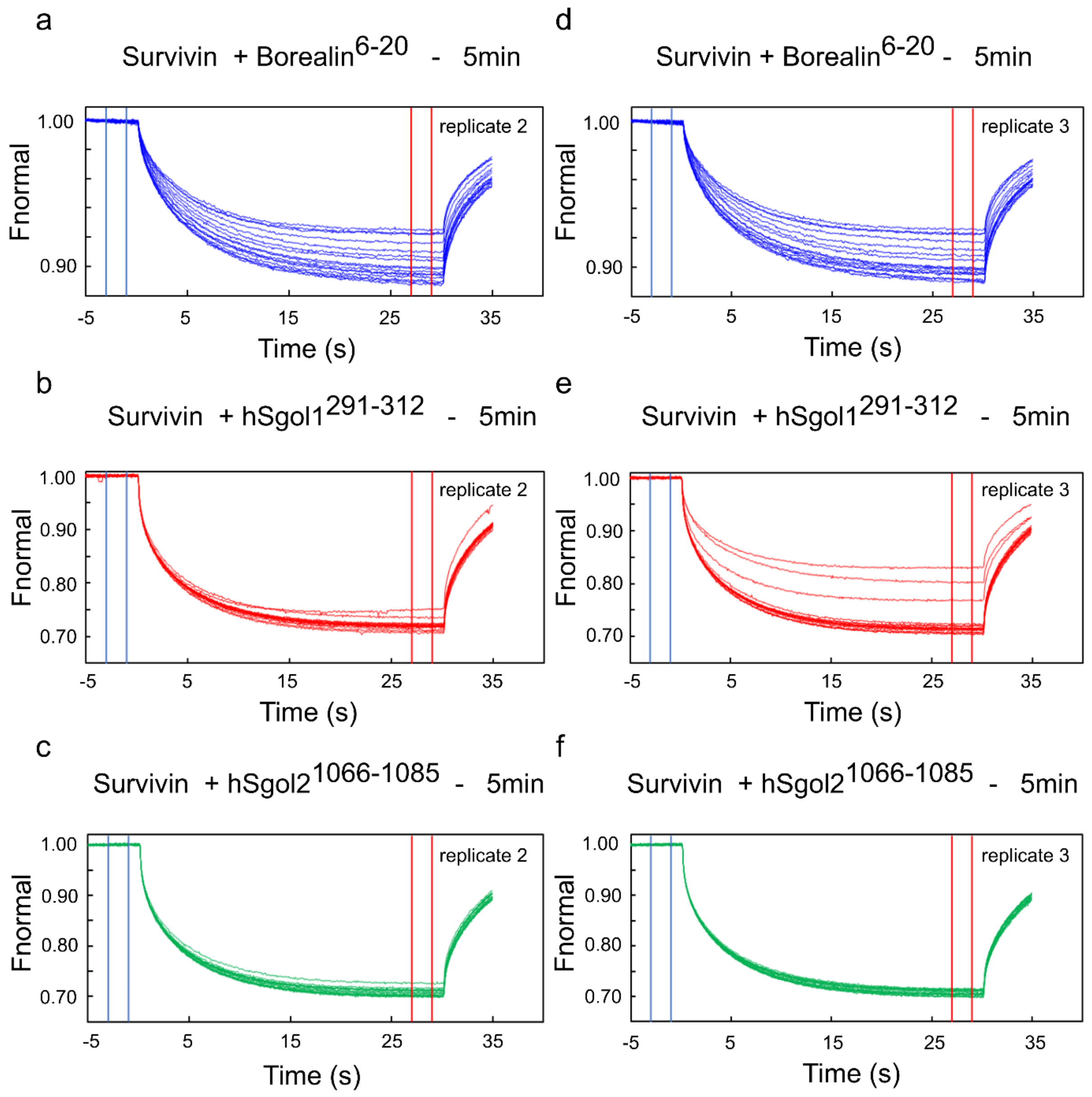

Figure S4. Primary thermophoresis data from interactions in buffer A and B after 5min incubation. Replicate 2 and 3 of the primary thermophoresis data from serial dilutions of Borealin$^{6-20}$ in buffer A (a and d), hSgol1$^{291-312}$ (b and e) and hSgol2$^{1066-1085}$ in buffer B (c and f) after 5min incubation together chemically labelled survivin. The cold and hot regions used to analyze the thermophoresis binding curves are represented by blue and red vertical lines, respectively.

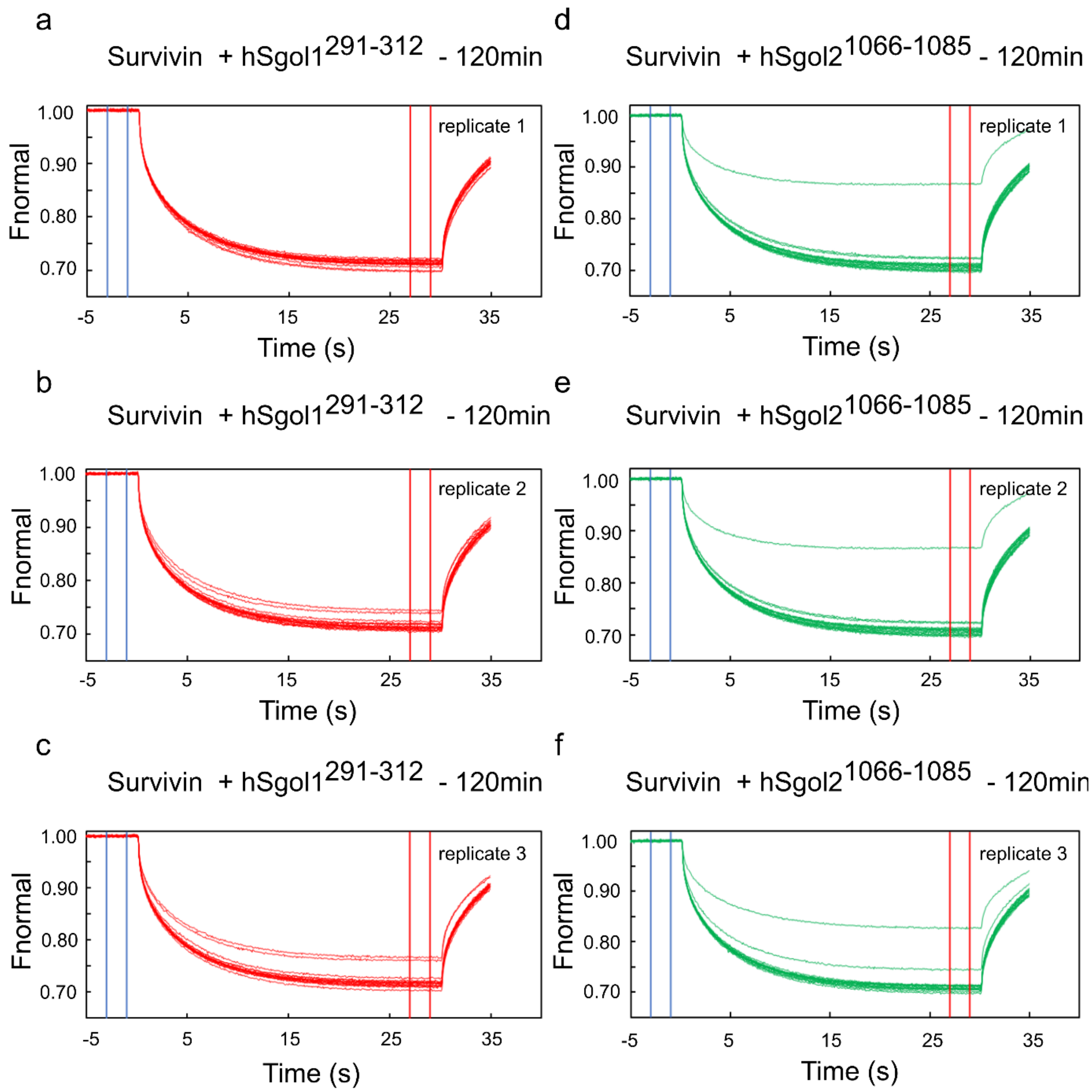

Figure S5. Primary thermophoresis data form interactions in buffer B after 120min incubation. Replicates of the primary thermophoresis data from serial dilutions of hSgol1$^{291-312}$ (a, b and c) and hSgol2$^{1066-1085}$ (d, e, and f) in buffer B after 120min incubation together with chemically labelled human survivin. The cold and hot regions used to analyze the thermophoresis binding curves are represented by blue and red vertical lines, respectively.

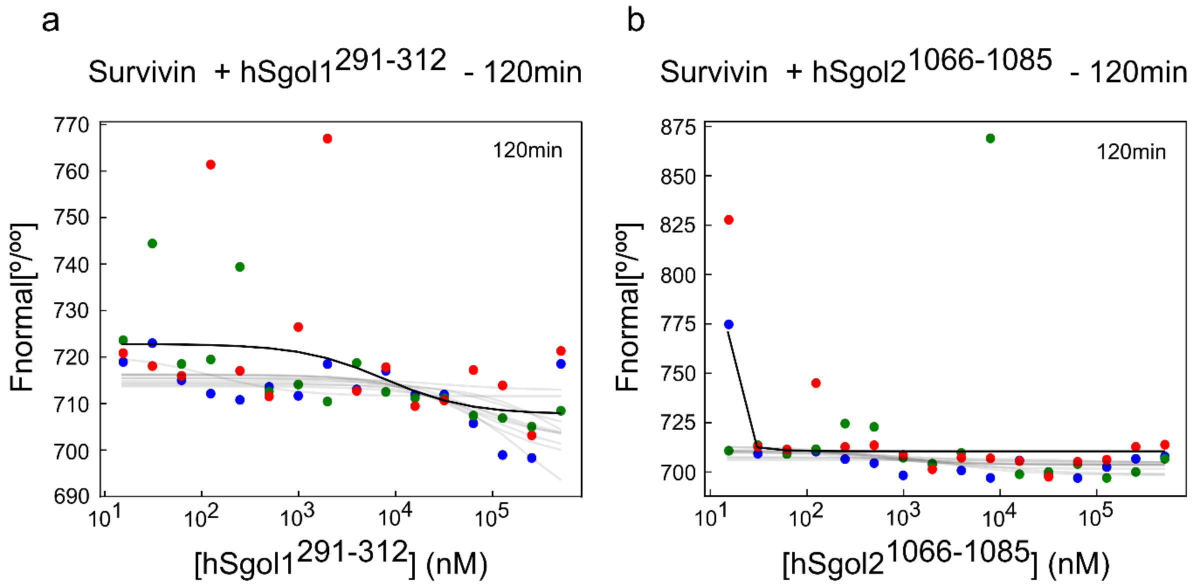

Figure S6. Bayesian analysis of experimental MST data of hSgol1$^{291\text{-}312}$ and hSgol2$^{1066\text{-}1058}$ in buffer B after 120min incubation. Thermophoresis binding curves of hSgol1$^{291\text{-}312}$ (a) and hSgol2$^{1066\text{-}1085}$ (b) interaction in buffer B with chemically labelled survivin for 120min incubation. The blue, green and red symbols represents measurements performed on three independent dilution series, respectively. Thick line represents the NLLSQ minimized binding curves, thin lines are ten random samples from the posterior distribution determined by the robust Bayesian procedure.

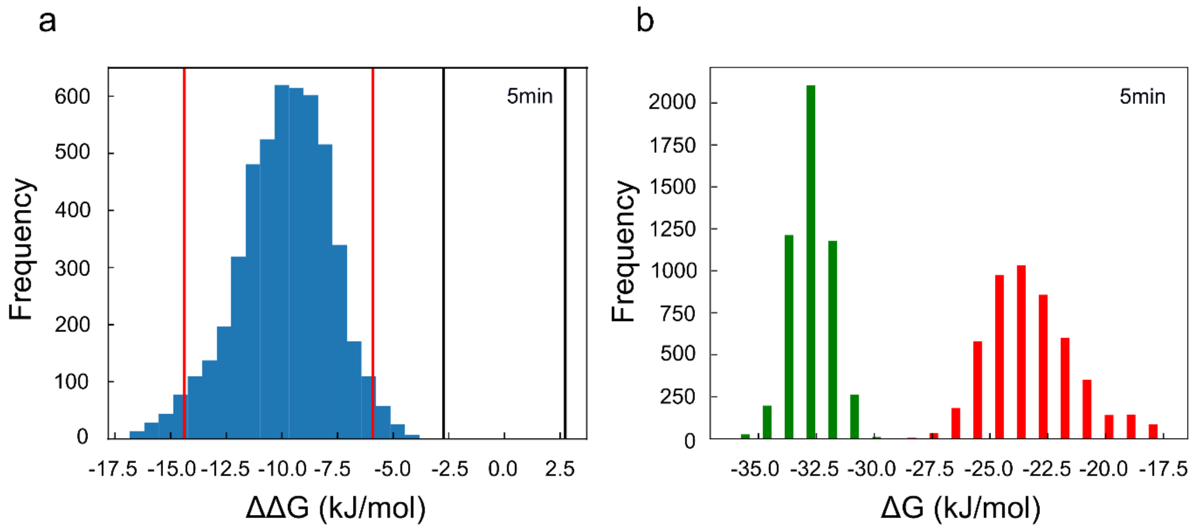

Figure S7. Binding free energy analysis of the hSgols and chemical labelled survivin interaction in buffer B. A *posteriori* binding affinity comparison between hSgol1$^{291\text{-}312}$ and hSgol2$^{1066\text{-}1085}$ with chemically labelled survivin in buffer B after incubation 5min. On the right side (a), it is represented the histogram of the estimated difference in binding free energy ($\Delta G_{hSgol2}{}^{291\text{-}312}$ - $\Delta G_{hSgol1}{}^{1066\text{-}1085}$). The black vertical lines represent the limits of the ROPE and the red vertical lines represent the limits of the highest density interval (HDI 95%). On the left side (b), it is represented the *a posteriori* distribution of binding free energy of hSgol1$^{291\text{-}312}$ (*red*) and S-Sgol2$^{1066\text{-}1085}$ (*green*) to human survivin.

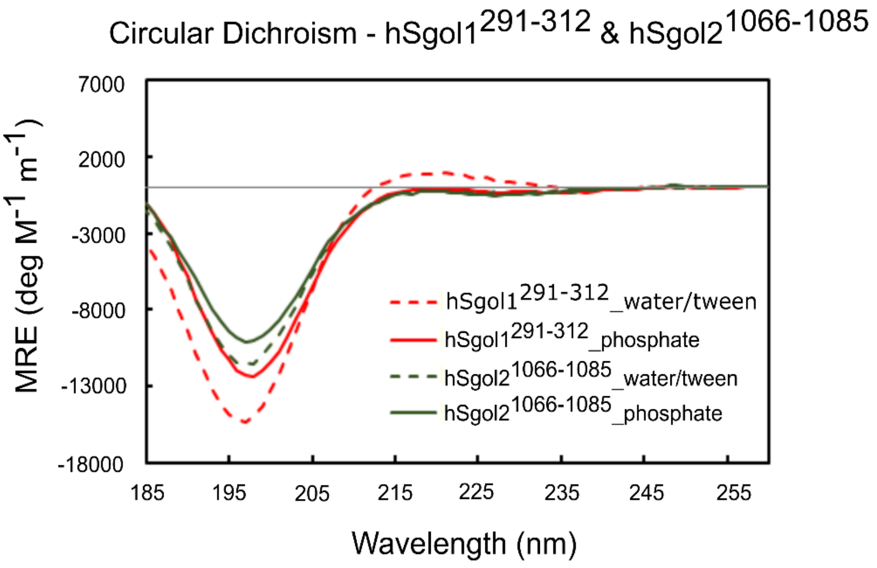

Figure S8. Circular dichroism spectroscopy of hSgol1$^{291\text{-}312}$ and hSgol2$^{1066\text{-}1085}$ peptides. Circular dichroism spectra of hSgol1$^{291\text{-}312}$ and hSgol2$^{1066\text{-}1085}$ in water with 0.05% Tween-20 (dashed lines) and in 20mM phosphate buffer pH8 (solid lines). hSgol1$^{291\text{-}312}$ and hSgol2$^{1066\text{-}1085}$ spectra are represented in red and green colors, respectively.

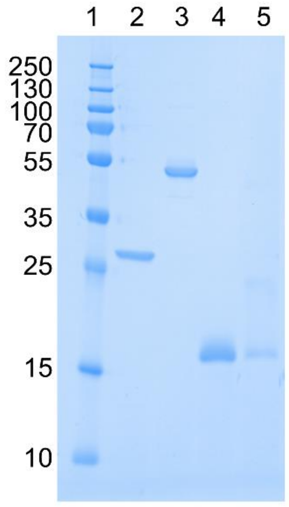

Figure S9. SDS – polyacrylamide gel electrophoresis of studied proteins. The numbers at the top represent the different samples loaded. The numbers at the left side correspond to the different molecular weights (kDa) of the protein ladder. 1. survivin (18.7kDa), 2. Chemically labelled survivin (approx. 18.7kDa).

References

1 Kruschke, J. K. Bayesian estimation supersedes the t test. *J. Exp. Psychol. Gen.* **142**, 573-603, doi:10.1037/a0029146 (2013).

2 Tiffany, M. L. & Krimm, S. Effect of temperature on the circular dichroism spectra of polypeptides in the extended state. *Biopolymers* **11**, 2309-2316, doi:10.1002/bip.1972.360111109 (1972).

3 Toal, S. & Schweitzer-Stenner, R. Local order in the unfolded state: conformational biases and nearest neighbor interactions. *Biomolecules* **4**, 725-773, doi:10.3390/biom4030725 (2014).

4 Scheuermann, T. H., Padrick, S. B., Gardner, K. H. & Brautigam, C. A. On the acquisition and analysis of microscale thermophoresis data. *Anal. Biochem.* **496**, 79-93, doi:10.1016/j.ab.2015.12.013 (2016).
